# Effects of insulin glargine U300 versus insulin degludec U100 on glycemic variability, hypoglycemia, and diet evaluated by continuous glucose monitoring in type 1 diabetes: a retrospective cross‐sectional study

**DOI:** 10.1002/kjm2.12909

**Published:** 2024-11-26

**Authors:** Pin‐Lun Tsai, Chia‐Hung Lin, Yu‐Yao Huang, Hsin‐Yun Chen, Yi‐Hsuan Lin

**Affiliations:** ^1^ Department of Medical Education Chang Gung Memorial Hospital Chiayi Taiwan; ^2^ Division of Endocrinology and Metabolism, Department of Internal Medicine Chang Gung Memorial Hospital Linkou Taiwan; ^3^ Department of Chinese Medicine, College of Medicine Chang Gung University Taoyuan Taiwan; ^4^ Department of Medical Nutrition Therapy Chang Gung Memorial Hospital Linkou Taiwan

**Keywords:** continuous glucose monitoring, glycemic index, long‐acting insulin, nutrient, time factor

## Abstract

The impacts of insulin degludec U100 (Deg‐100) and insulin glargine U300 (Gla‐300) on glycemic variability (GV) in patients with type 1 diabetes, as well as the impact of major nutrient components on GV in these patients, remain unclear. This was an observational, cross‐sectional, retrospective study. Type 1 diabetes mellitus patients treated with either Deg‐100 or Gla‐300 as basal insulin were enrolled. After the participants underwent continuous glucose monitoring, GV indices and major nutrient components were analyzed. Forty patients with type 1 diabetes were enrolled, and 20 participants used Deg‐100, and 20 used Gla‐300. There was no significant difference in major nutrient components between the two groups. Better GV indices of standard deviation, coefficient of variation, mean amplitude of glycemic excursion, AUC_*n*_, *M*‐value, CONGA1, CONGA2, and CONGA4 were noted in the Gla‐300 group versus Deg‐100 group. Compared with patients who received once‐daily injection in the morning (QD), Deg‐100 administration once daily at bedtime (HS) yielded a higher low blood glucose index during both day and nocturnal periods, indicating a higher risk of hypoglycemic events. By contrast, there were significantly lower levels of CONGA1, CONGA2, and CONGA4 during insulin Gla‐300 QD administration than during HS administration, indicating a lower GV of a short interval. In this real‐world study involving type 1 diabetes patients, Gla‐300 appears to offer more stable glucose variability than Deg‐100. Administering once‐daily injections could lower the risk of hypoglycemia in the Deg‐100 group and minimize GV in the Gla‐300 group compared to bedtime injections.

## 1 INTRODUCTION

Current evidence suggests that HbA1c, in addition to glycemic variability (GV), could affect the possibility of hypoglycemic events and the development of diabetes complications.^1^, ^2^, ^3^, ^4^, ^5^, ^6^ For patients with type 1 diabetes, who require life‐long insulin administration, stable GV control is the main goal for clinical practitioners.

Insulin degludec U100 (Deg‐100) and insulin glargine U300 (Gla‐300) are the second‐generation long‐acting insulin analogues in current clinical practice and have proven better performance not only in day‐to‐day GV but also in within‐day GV than Gla‐100.^7^, ^8^, ^9^, ^10^ Some studies were conducted to investigate the efficacy and safety of the two types of insulin analogues for type 2 diabetes, which represented the vast majority. However, limited studies on type 1 diabetes patients have been conducted in glucose clamp settings.^11^, ^12^ EDITION series studies revealed a lower hypoglycemia risk with Gla‐300 than with insulin glargine U100.^13^, ^14^, ^15^ Meta‐analyses have reported that Deg‐100 could yield a reduction in severe hypoglycemic events^16^; however, another randomized controlled trial suggested that Deg‐100 could result in greater time below the target range and less time above the target range, reflecting the unsettlement of this issue.^17^

This was a pilot study in which CGM was performed to compare GV and correlation with major nutrient components between the two second‐generation basal insulin analogues: insulin Gla‐300 and insulin Deg‐100. Information regarding the injection time of basal insulin, daily nutrient intake, and after‐CGM follow‐up was also obtained to gain other insights.

## 2 MATERIALS AND METHODS

### 2.1 Study design

This was an observational, cross‐sectional, retrospective study conducted at Linkou Chang Gung Memorial Hospital, Taiwan. In this study, we examined the impact of Deg‐100 and Gla‐300 on the GV of patients with type 1 diabetes in real‐world practice. Type 1 diabetes mellitus was diagnosed according to low C‐peptide level, positive antibodies, and positive glucagon‐stimulation test and applied catastrophic illness approved by Taiwan National Health Insurance. Major nutrient components and the timing of basal insulin injection were both recorded to elucidate the effects of two different basal insulin levels and nutrient components on GV.

We reviewed medical records of type 1 diabetes mellitus patients and those treated with either Deg‐100 or Gla‐300 as basal insulin and received continuous glucose monitoring (CGM) from March 2019 to December 2021 were screened. Further inclusion criteria were older than 18 years old, diagnosis of type 1 diabetes for at least 6 months, and ambulatory status. As routine clinical practice, the participants were asked not to change their diet and exercise habits and to record a detailed food diary during the CGM period. The exclusion criteria were a recent history of substance abuse, serious cardiovascular disorders, ongoing influenza, autoimmune diseases, or just surgery within one month. Finally, a total of 40 participants were corresponded. This study was approved by the Institutional Review Board (IRB) and ethics committees of Chang Gung Memorial Hospital (CGMH) (IRB No. 201701492B0). The IRB waived the requirement for obtaining informed consent. The research subjects’ confidentiality was maintained according to the requirements of the IRB of CGMH (Taipei, Taiwan).

### 2.2 Diet records

During the CGM period, participants meticulously documented their dietary intake in a comprehensive food diary. This included the cooking methods, as well as the types and quantities of food and beverages consumed for breakfast, lunch, dinner, and snacks. A certified dietitian then thoroughly analyzed these diaries to accurately calculate the primary nutritional components such as carbohydrates, proteins, and fats.

### 2.3 Glucose monitoring

In this study, we utilized two CGM systems: CGMS Gold (MiniMed CGMS MMT‐7102‐W, Medtronic, Inc., Northridge, CA, USA) and Guardian Connect CGM system (Medtronic, Inc., Northridge, CA, USA). The CGM devices, recording for a period ranging between 5 and 10 days (with an average of 7.2 days), were subcutaneously inserted either in the abdomen or arm. These sensors measured the glucose levels in the interstitial fluid every 10 s, averaging and recording the data every 5 min. Both devices had a glucose detection range of 40–400 mg/dL, with values beyond this range being recorded as either 400 or 40 mg/dL, respectively.

### 2.4 Outcome measures

Participants were divided into two groups depending on the type of basal insulin analogues used: one group received Deg‐100 and the other group received Gla‐300. Each group was divided into two subgroups based on the timing of basal insulin injection (once daily in the morning [QD] or once daily bedtime [HS]).

Raw data were extracted from either the MiniMed Solutions CGM sensor or Guardian Connect CGM sensor using CareLink™ iPro software (Medtronic, Inc., Northridge, CA, USA) after study completion. Several parameters were calculated, including standard deviation (SD), percentage coefficient of variation (%CV), and mean amplitude of glycemic excursion (MAGE).^18^ The area under the curve (AUC) of a glucose level of >180 and <70 mg/dL were calculated and presented as AUC_180_ and AUC_70_, representing the hyperglycemic and hypoglycemic exposure, respectively, whereas AUC_*t*_ and AUC_*n*_, respectively, reflected the total and normal (70–180 mg/dL) AUCs of glucose levels.^19^ The risks of hypoglycemic and hyperglycemic events were calculated as the low blood glucose index (LBGI) and high blood glucose index (HBGI), respectively.^20^ *M*‐value was used as a parameter to assess GV.^21^ Intra‐day GV was evaluated by calculating continuous overlapping net glycemic action (CONGA). CONGA_*n*_ represents the differences in all recorded SDs between the current observation and the value recorded in the *n*‐h period.^22^ Time above range (TAR), time in range (TIR), and time below range (TBR) were obtained from CGM recordings, which reflect the percentage of time when the glucose concentration of the interstitial fluid was above 180 mg/dL, within the range of 70–180 mg/dL or below 70 mg/dL.^23^

### 2.5 After CGM follow‐up

Two to three months after the first CGM recording, these participants returned to our outpatient department, where HbA1c was reexamined, and the daily insulin dose was recorded. The type and dosage of medications, including basal insulin, and bolus insulin, were documented and compared with those before CGM recording.

### 2.6 Statistical analysis

All data analyses were performed with the Statistical Package for the Social Sciences, version 26.0 (IBM Corp., Armonk, NY, USA). Differences in continuous variables between the two groups were calculated using Mann–Whitney U tests owing to limited sample size, and we assumed that the test did not follow a normal distribution. Correlations between two continuous variables were analyzed using Spearman's correlation coefficient analysis. Nominal variables between the groups were analyzed using McNemar's chi‐square test. A *p*‐value of <0.05 was considered to indicate statistical significance.

## 3 RESULTS

### 3.1 Demographic characteristics

A total of 40 patients (30 women and 10 men) who were diagnosed with type 1 diabetes and received CGM from March 2019 to December 2021 at Linkou Chang Gung Memorial Hospital, Taiwan, were enrolled in this study. The most common reason for performing CGM was high‐glucose excursion. The ages of participants varied from 18.1 to 64.7 years. The study consisted of two distinct groups, each comprising 20 participants: one group received Deg‐100, while the other was administered Gla‐300 as their basal insulin therapy. The detailed demographic characteristics of patients are presented in Table 1. There was no significant difference between the groups regarding basic profiles, including age, sex, duration of type 1 diabetes, and dosages of both basal and bolus insulin. Concerning daily dietary consumption, participants had an average total caloric intake of 1446.6 kcal per day. The intake of carbohydrates, proteins, and fats averaged 2.78, 0.99, and 0.99 g/kg/day, respectively, per kilogram of body weight. Notably, there was no significant difference in these nutritional intakes between the two groups.

**TABLE 1:** Demographic and major nutrient components characteristics of participants.

	Total study population	Received insulin degludec U100	Received insulin glargin U300	*p*
	(*n* = 40)	(*n* = 20)	(*n* = 20)	
Age, years	33.68 [29.63, 49.98]	32.99 [30.37, 51.19]	34.45 [28.86, 45.31]	0.698
Sex, male, *n* (%)	10 (25.0)	6 (30.0)	4 (20.0)	0.465
Duration of CGM, days	7 [7, 8]	7 [7, 8]	7 [6.25, 8]	0.738
Body weight, kg	59.5 [51.25, 68]	60.5 [50.75, 67.75]	58.95 [51.25, 70.25]	1.000
BMI, kg/m^2^	22.74 [20.42, 25.31]	22.74 [21.81, 25.19]	22.49 [20.34, 25.84]	0.758
Duration of disease, years	13 [6, 20]	12.5 [5.25, 21.5]	13 [8, 20]	0.835
Total daily insulin dose, U (divided by body weight, U/kg)	43 [33, 58]	38.5 [31.25, 60]	46.5 [36, 57.5]	0.620
	(0.73 [0.56, 0.94])	(0.64 [0.54879679, 1.11672794])	(0.76 [0.62, 0.93])	(0.583)
Basal daily insulin dose, U (divided by body weight, U/kg)	15.5 [11.25, 20.75]	18.5 [12, 20.75]	15 [10, 21]	0.445
	(0.27 [0.22, 0.34])	(0.29 [0.22, 0.34])	(0.26 [0.18, 0.32])	(0.414)
HbA1c before CGM, % (mmol/mol)	8.3 [7.3, 9]	8.35 [7.9, 9.3]	7.9 [7.2, 9]	0.258
	(67.22 [56.29, 74.87])	(67.77 [62.85, 78.15])	(62.85 [55.2, 74.87])	(0.258)
Nutrient composition per day				
Carbohydrate, % (g)	46.08 [43.34, 50.23]	47.22 [44.61, 51.03]	44.81 [41.07, 49.87]	0.242
	(164.51 [142.50, 193.97])	(164.51 [143.09, 201.73])	(164.58 [119.76, 189.3])	(0.698)
Protein, % (g)	15.89 [14.68, 17.97]	15.32 [14.08, 17.57]	16.57 [15.01, 18.34]	0.108
	(58.06 [48.03, 67.30])	(59.72 [48.65, 66.89])	(56.14 [44.61, 75.63])	(0.968)
Fat, % (g)	37.41 [32.92, 40.92]	37.40 [32.23, 39.99]	37.63 [33.64, 42.05]	0.547
	(60.94 [47.03, 72.61])	(63.17 [47.38, 72.40])	(54.48 [44.78, 77.26])	(0.779)
Average daily carbohydrates per body weight, g/kg	2.66 [2.50, 3.01]	2.67 [2.58, 3.01]	2.62 [2.2, 3.05]	0.461
Average daily protein per body weight, g/kg	0.97 [0.77, 1.11]	0.95 [0.76, 1.15]	0.97 [0.83, 1.09]	0.968
Average daily fat per body weight, g/kg	0.99 [0.79, 1.20]	1.00 [0.80, 1.18]	0.95 [0.79, 1.2]	0.659
Average daily calories, kcal	1405.77 [1155.53, 1726.51]	1431.10 [1223.78, 1718.39]	1319.23 [1142.81, 1756.66]	0.799
Average daily calories per body weight, kcal/kg	23.24 [20.72, 27.25]	23.50 [21.16, 25.98]	22.98 [18.01, 28.34]	0.547

*Note*: Data of age, duration of CGM, body weight, body mass index, disease duration, insulin dose, and nutrient composition are presented as median [Q1, Q3]. Continuous variants were analyzed using the Mann–Whitney U test, and nominal variants were analyzed using McNemar's chi‐square test.

Abbreviations: BMI, body mass index; CGM, continuous glucose monitoring; HbA1c, glycated hemoglobin A1c; *Q*, quartile.

Participants in both the Deg‐100 and Gla‐300 groups were further divided into two subgroups based on the timing of basal insulin injection. Within the group of participants administered Deg‐100, 13 of them used it QD, whereas the remaining 7 received it HS. Among those treated with Gla‐300, 16 participants were administered QD injections, 3 received their injections HS, and 1 participant was on a regimen of twice‐daily injections. Participants who were administered Gla‐300 twice daily were excluded from the analysis comparing the effects of basal insulin administered at different times. Comprehensive demographic details of the remaining participants are provided in Supplementary Table 1, showing no significant differences between these subgroups.

### 3.2 CGM index

The computerized analysis evaluated GV indices of both groups over three different periods: all‐day (00:00–24:00), nocturnal (00:00–06:00), and diurnal (06:00–24:00), as detailed in Table 2. There were no significant differences of TIR, TAR, and TBR between the two groups. Moreover, the Gla‐300 group demonstrated superior GV indices compared to the Deg‐100 group, including SD, CV, MAGE, AUC*n*, *M*‐value, CONGA1, CONGA2, and CONGA4.

**TABLE 2:** Results of computerized glycemic variability index.

	Total study population	Received insulin degludec U100	Received insulin glargin U300	*p*
	(*n* = 40)	(*n* = 20)	(*n* = 20)	
All day period (00:00–24:00)				
SD, mg/dL	56.9 [45.74, 66.85]	63.28 [53.23, 73.5]	54.55 [40.32, 59.31]	0.009^a^
CV	0.33 [0.3, 0.39]	0.36 [0.32, 0.41]	0.32 [0.28, 0.37]	0.033^a^
MAGE, mg/dL	138.06 [115.67, 166.83]	156.56 [136.33, 171.88]	126.57 [93.41, 140.12]	0.006^a^
AUC_*t*_, mg/dL	46485.01 [38906.84, 54248.73]	49304.9 [39131.46, 58350]	43318.29 [38600.95, 49371.98]	0.192
AUC_180_, mg/dL	9223.37 [9223.37, 9223.37]	9223.37 [9223.37, 9223.37]	9223.37 [9223.37, 9223.37]	0.056
AUC_*n*_, mg/dL	19063.9 [14428.04, 24484.45]	15532.68 [13553.75, 20621]	22847.25 [16199.45, 27350.15]	0.021^a^
AUC_70_, mg/dL	730.22 [254.4, 1388.87]	812.08 [309.25, 1371.39]	512.2 [105.45, 1419.04]	0.277
LBGI, mg/dL	0.91 [0.4, 1.84]	0.99 [0.5, 2.9]	0.8 [0.32, 1.72]	0.327
HBGI, mg/dL	7.49 [4.35, 13.64]	10.83 [4.96, 16.6]	6.94 [3.92, 9.9]	0.102
*M*‐value, mg/dL	24.32 [18.61, 39.74]	32.44 [20.39, 42.56]	22.3 [14.35, 28.09]	0.033^a^
CONGA1, mg/dL	42.4 [34.99, 48.19]	45.97 [38.23, 51.05]	39.28 [29.4, 45.08]	0.020^a^
CONGA2, mg/dL	61.6 [50.81, 74.19]	67.72 [56.63, 80.07]	57.27 [42.54, 65.4]	0.028^a^
CONGA4, mg/dL	79.16 [62.89, 91.12]	84.4 [72.07, 96.69]	74.77 [56.22, 86.75]	0.043^a^
TAR, %	37.1 [24.15, 55.7]	46.92 [25.13, 59]	30.5 [24.15, 47.5]	0.192
TIR, %	54.5 [39, 68.1]	46.4 [38, 60.06]	66 [46.96, 69]	0.060
TBR, %	3.1 [1.04, 7.6]	4.17 [1.81, 12.03]	3 [0.21, 7.2]	0.174
Nocturnal period (00:00–06:00)				
SD, mg/dL	29.93 [21.93, 34.98]	31.41 [24.44, 34.7]	25.58 [18.53, 36.25]	0.231
CV	0.19 [0.13, 0.24]	0.18 [0.16, 0.22]	0.19 [0.11, 0.24]	0.947
MAGE, mg/dL	69.17 [48.82, 83.48]	74.88 [53.6, 83.21]	60.35 [41.43, 101.46]	0.398
AUC_*t*_, mg/dL	11101.33 [8782.79, 14417.83]	13608.54 [9346.53, 16451.71]	10706.14 [8433.78, 12865.19]	0.091
AUC_180_, mg/dL	5558.3 [1933.44, 9223.37]	9223.37 [3925.69, 9223.37]	3943.23 [622.72, 7624.25]	0.033
AUC_*n*_, mg/dL	4805.6 [2512.18, 6273.31]	3402.54 [1939.81, 5236.67]	6081.79 [3400.43, 7171.83]	0.005^a^
AUC_70_, mg/dL	145.14 [0, 494.13]	137.03 [0, 559.6]	145.14 [0, 494.13]	0.862
LBGI, mg/dL	0.96 [0.19, 2.32]	0.96 [0.06, 2.76]	1.08 [0.28, 2.32]	0.947
HBGI, mg/dL	7.15 [2.43, 15.23]	11.96 [4.81, 17.86]	5.04 [1.85, 9.08]	0.017^a^
*M*‐value, mg/dL	18.86 [9.85, 34.39]	24.5 [18.61, 44.61]	11.05 [8.22, 20.07]	0.003^a^
CONGA1, mg/dL	21.29 [15.96, 26.24]	22.29 [18.73, 26.05]	17.36 [10.33, 27.74]	0.201
CONGA2, mg/dL	26.84 [20.29, 34.6]	28.67 [23.87, 32.1]	22.69 [12.74, 35.55]	0.157
CONGA4, mg/dL	21.67 [15.26, 28.17]	23.55 [20.65, 25.87]	18.62 [9.69, 30.11]	0.149
Diurnal period (06:00–24:00)				
SD, mg/dL	55.69 [44.08, 66.49]	58.71 [52.11, 74.3]	52.43 [38.38, 58.92]	0.035^a^
CV	0.33 [0.29, 0.38]	0.36 [0.31, 0.41]	0.29 [0.26, 0.35]	0.009^a^
MAGE, mg/dL	123.97 [108.3, 154.53]	137.83 [118.15, 175.92]	115.9 [94, 129.59]	0.011^a^
AUC_*t*_, mg/dL	34380.33 [30421.58, 39708.71]	36562.85 [29145.75, 44377.11]	33152.88 [31061.38, 36990.23]	0.495
AUC_180_, mg/dL	9223.37 [9223.37, 9223.37]	9223.37 [9223.37, 9223.37]	9223.37 [9211.25, 9223.37]	0.183
AUC_*n*_, mg/dL	13790.19 [11666.59, 18897.46]	12956.79 [10744.18, 16583.84]	16202.88 [12764.52, 20515.88]	0.030^a^
AUC_70_, mg/dL	545.17 [147.69, 1048.77]	588.92 [269.98, 1136.78]	350.05 [13.75, 770.08]	0.114
LBGI, mg/dL	0.94 [0.4, 1.79]	1.04 [0.53, 2.86]	0.55 [0.24, 1.33]	0.121
HBGI, mg/dL	7.96 [4.33, 12.43]	11.05 [4.42, 15.83]	7.64 [4.21, 10.5]	0.221
*M*‐value, mg/dL	24.97 [17.27, 36.88]	30.11 [18.85, 38.84]	23.22 [14.1, 30.73]	0.091
CONGA1, mg/dL	44.58 [37.61, 52.42]	49.98 [40.06, 55.73]	40.38 [33.25, 49.11]	0.035^a^
CONGA2, mg/dL	64.49 [53.57, 77.27]	72.07 [60.51, 86.55]	61.39 [48.09, 75.03]	0.060
CONGA4, mg/dL	80.14 [58.19, 94.79]	82.12 [70.87, 105.09]	75.15 [53.66, 91.03]	0.174

*Note*: Data of each glycemic variability index are presented as median [Q1, Q3]. Continuous variants were analyzed by Mann–Whitney U test.

Abbreviations: AUC, area under the curve; CONGA, continuous overlapping net glycemic action; CV, coefficient of variation; HBGI, high blood glucose index; LBGI, low blood glucose index; MAGE, mean amplitude of glycemic excursions; M‐value, weighted average of glucose value; *Q*, quartile; TAR, time above range; TIR, time in range; TBR, time below range; SD, standard deviation.

^a^ Denotes a *p*‐value of <0.05.

Spearman's correlation coefficient analysis (Table 3) revealed significant correlations between GV indices (SD, CV, AUC*n*, and TIR) and variables such as age, body mass index, diabetes duration, baseline HbA1c, total calorie intake, and nutrient components. Notably, a higher percentage of daily protein intake was significantly correlated with AUCn and TIR (*r* = 0.429, 0.336, respectively, *p*<0.05). Similarly, fat intake per body weight was significantly related to SD, AUC*n*, and TIR (*r* = 0.374, −0.377, and −0.436, respectively, *p*<0.05). Caloric intake per body weight showed a positive correlation with SD (*r* = 0.331, *p*<0.05) and a negative correlation with TIR (*r* = −0.358, *p*<0.05).

**TABLE 3:** Correlation of SD, CV, AUC_*n*_, and time in range with age, BMI, diabetes duration, HbA1c value before CGM study, and percentages of carbohydrate, protein, and fat intake per day.

Parameter	SD (mg/dL)		CV (%)		AUC_*n*_ (mg/dL)		Time in range (%)	
	Correlation coefficient	*p*	Correlation coefficient	*p*	Correlation coefficient	*p*	Correlation coefficient	*p*
Age, years	−0.157	0.333	−0.145	0.371	0.069	0.670	0.125	0.443
BMI, kg/m^2^	0.091	0.576	0.087	0.595	−0.094	0.562	−0.053	0.743
Diabetes duration, years	0.275	0.090	0.180	0.273	−0.223	0.171	−0.188	0.251
HbA1c, %	0.284	0.079	0.111	0.499	−0.239	0.143	−0.257	0.114
Carbohydrate, %	−0.056	0.732	0.119	0.465	0.063	0.700	0.132	0.415
Protein, %	−0.243	0.131	−0.175	0.279	0.429	0.006^a^	0.336	0.034^a^
Fat, %	0.169	0.297	−0.009	0.954	−0.270	0.092	−0.304	0.056
Carbohydrate (g)/BW (kg)	0.258	0.108	0.002	0.990	−0.250	0.120	−0.276	0.085
Protein (g)/BW (kg)	0.206	0.202	−0.067	0.680	−0.052	0.752	−0.165	0.308
Fat (g)/BW (kg)	0.374	0.017^a^	0.064	0.694	−0.377	0.016^a^	−0.436	0.005^a^
Calories (kcal)/BW (kg)	0.331	0.037^a^	−0.017	0.918	−0.288	0.072	−0.358	0.023^a^

Abbreviations: AUC, area under the curve; BMI, body mass index; BW, body weight; CGM, continuous glucose monitoring; CV, coefficient of variation; HbA1c, glycated hemoglobin; SD, standard deviation.

^a^ Spearman's correlation is significant at the 0.05 level (2‐tailed).

Table 4 presents GV indices for subgroups based on the timing of basal insulin injections. In the Deg‐100 group, the LBGI was significantly lower in participants using QD injections compared to HS injections. In the Gla‐300 group, CONGA1, CONGA2, and CONGA4 indices were lower in the QD subgroup than in those using HS injections.

**TABLE 4:** Results of computerized glycemic variability index between users of insulin degludec U100 and insulin glargine U300 in the morning and at bedtime.

	Received insulin degludec U100 (*n* = 20)			Received insulin glargin U300 (*n* = 20)		
	Bedtime (*n* = 13)	Morning (*n* = 7)	*p*	Bedtime (*n* = 16)	Morning (*n* = 3)	*p*
All day period (00:00–24:00)						
SD, mg/dL	61.76 [51.43, 80.43]	64.92 [61.16, 69.6]	0.757	54.64 [41.58, 59.31]	38.87 [36.84, ‐]	0.064
CV, %	0.39 [0.33, 0.44]	0.32 [0.3, 0.35]	0.081	0.32 [0.28, 0.38]	0.31 [0.23, ‐]	0.421
MAGE, mg/dL	157.83 [125.65, 183.67]	155.28 [137.18, 169.83]	1.000	126.57 [102.53, 155.44]	89.42 [89.35, ‐]	0.359
AUC*t*, mg/dL	48484.5 [37719.98, 53068.46]	57620.75 [47047.42, 64140.1]	0.097	43318.29 [39432.45, 49371.98]	37546 [31916.38, ‐]	0.211
AUC180, mg/dL	9223.37 [9223.37, 9223.37]	9223.37 [9223.37, 9223.37]	0.081	9223.37 [9223.37, 9223.37]	7844.25 [7489, ‐]	0.171
AUC*n*, mg/dL	18240.7 [13818.83, 22812.8]	15417.25 [9732.5, 18728.4]	0.393	22847.25 [13964.31, 26579.08]	27579.2 [19455.63, ‐]	0.303
AUC70, mg/dL	1006.33 [667.5, 2105.13]	407.5 [267.8, 784.6]	0.081	605.62 [171.05, 1419.04]	0 [0, ‐]	0.559
LBGI, mg/dL	1.39 [0.91, 3.68]	0.5 [0.33, 0.83]	0.030^a^	0.89 [0.32, 1.72]	0.82 [0.04, ‐]	0.958
HBGI, mg/dL	9.86 [3.82, 14.89]	14.59 [7.67, 18.38]	0.115	7.12 [4.54, 11.15]	2.81 [2.46, ‐]	0.109
*M*‐value, mg/dL	27.38 [18.65, 44.75]	36.9 [24.88, 42.34]	0.438	22.7 [15.54, 34.63]	14.3 [10.89, ‐]	0.138
CONGA1, mg/dL	45.94 [37.18, 51.8]	45.99 [40.06, 48.22]	1.000	40.27 [34.06, 47.21]	24.81 [19.18, ‐]	0.008^a^
CONGA2, mg/dL	66.74 [52.06, 80.81]	68.69 [65.38, 73.49]	0.757	58.62 [50.81, 72.17]	39.81 [30.22, ‐]	0.014^a^
CONGA4, mg/dL	84.23 [69.06, 109.26]	84.58 [79.18, 91.67]	0.817	75.86 [60.95, 86.75]	50.62 [30.32, ‐]	0.023^a^
TAR, %	45.17 [18.2, 52.5]	60 [40, 73.2]	0.056	29.6 [24.15, 52.05]	31 [13, ‐]	0.634
TIR, %	50 [38.5, 68.5]	39 [25.2, 54]	0.157	66 [42.7, 69.55]	69 [65, ‐]	0.421
TBR, %	5 [3.5, 15.67]	2 [1.6, 3.2]	0.081	3 [1.04, 7.2]	0 [0, ‐]	0.634
Nocturnal period (00:00‐06:00)						
SD, mg/dL	30.62 [22.5, 32.75]	35.1 [29.24, 39.6]	0.081	27.47 [21.65, 36.25]	12.48 [3.34, ‐]	0.085
CV	0.19 [0.16, 0.23]	0.16 [0.13, 0.22]	0.699	0.2 [0.12, 0.24]	0.07 [0.04, ‐]	0.064
MAGE, mg/dL	71.5 [48.85, 82.46]	81.1 [58.92, 113.6]	0.183	61.23 [47.31, 104.83]	39.6 [11, ‐]	0.171
AUC*t*, mg/dL	11260.5 [8506.38, 14468.66]	14931.58 [11813.08, 17685.13]	0.081	10706.14 [8433.78, 13536.84]	10143.38 [6297, ‐]	0.559
AUC180, mg/dL	5860.83 [2196.63, 9223.37]	9223.37 [8853, 9223.37]	0.067	3435.99 [622.72, 9223.37]	4482.63 [0, ‐]	0.793
AUC*n*, mg/dL	4270.75 [2629.1, 5462.21]	2409.25 [1258.5, 3373.08]	0.056	6081.79 [2920.94, 7394.85]	6297 [5381.5, ‐]	1.000
AUC70, mg/dL	200 [11.67, 904.96]	16.17 [0, 307.58]	0.157	244.1 [0, 525.03]	0 [0, ‐]	0.303
LBGI, mg/dL	1.19 [0.58, 5.25]	0.17 [0, 1.06]	0.046^a^	1.19 [0.28, 2.43]	1.43 [0, ‐]	0.559
HBGI, mg/dL	9.14 [2.58, 15.1]	17.78 [10, 23.12]	0.081	4.76 [1.85, 11.09]	4.91 [0, ‐]	0.634
*M*‐value, mg/dL	23.54 [16.74, 42.98]	35.74 [19.26, 45.11]	0.588	12.59 [8.22, 27.9]	9.79 [3.17, ‐]	0.254
CONGA1, mg/dL	22.65 [17.41, 25.47]	21.68 [18.85, 39.07]	0.536	19.4 [12.77, 27.74]	9.56 [4.46, ‐]	0.109
CONGA2, mg/dL	26.09 [21.83, 31.6]	29.37 [27.59, 54.09]	0.135	23.81 [16.52, 35.55]	11.55 [5.9, ‐]	0.138
CONGA4, mg/dL	24.41 [18.45, 26.92]	22.69 [20.62, 26.17]	1.000	21.05 [12.08, 34.45]	9.02 [1.56, ‐]	0.109

*Note*: Data of each glycemic variability index are presented as median [Q1, Q3]. Continuous variants were analyzed by Mann–Whitney U test.

Abbreviations: AUC, area under the curve; CONGA, continuous overlapping net glycemic action; CV, coefficient of variation; HBGI, high blood glucose index; LBGI, low blood glucose index; *M*‐value, weighted average of glucose value; MAGE, mean amplitude of glycemic excursions; TAR, time above range; TIR, time in range; TBR, time below range; SD, standard deviation.

^a^ Denotes a *p*‐value of <0.05.

### 3.3 After CGM follow‐up

Three months following the CGM session, we recorded and analyzed both HbA1c levels and daily as well as basal insulin doses across the two groups, with the findings detailed in Supplementary Table 2. Notably, there were no significant differences in HbA1c levels or in the total and basal insulin doses before and after the CGM period.

## 4 DISCUSSION

According to current study, Gla‐300 led to a more stable GV than Deg‐100. Furthermore, Gla‐300 QD injection provided lower GV than HS injection, and Deg‐100 QD injection had lower incidence of hypoglycemia events than HS injection.

Several studies have indicated that Deg‐100 possesses a higher glucose‐lowering potency compared to Gla‐300.^11^, ^24^ However, opinions on GV differ. The BRIGHT study, a randomized trial, found that insulin‐naïve type 2 diabetes patients achieved similar within‐day plasma glucose variability with both Deg‐100 and Gla‐300, but a higher rate of nocturnal hypoglycemia episodes was observed during the titration period with Deg‐100.^24^ The INEOX‐Plus study noted comparable efficacy and safety between the two, except for a significantly lower annual hypoglycemia event rate in Gla‐U300 users.^25^ Bailey et al. found that at a daily morning dosage of 0.4 U/kg, Gla‐300 exhibits steadier pharmacodynamic profiles with reduced daily variability and more uniform pharmacokinetic distribution than Deg‐100.^26^ Conversely, some research reported lower GV and hypoglycemia episodes with Deg‐100,^27^, ^28^ while the InRange trial, using CGM to compare these basal insulins, revealed similar TIR and hypoglycemia risk.^29^ Another meta‐analysis found no significant difference in glucose control and hypoglycemia risk between Deg‐100 and Gla‐300.^30^ In contrast, the research conducted by Lucidi et al. focused on the pharmacokinetics (PK) of clinical doses of these second‐generation basal insulins, revealing that Gla‐300's PK was more stable and evenly distributed over 24 h, while Deg‐100 exhibited a 22% greater peak activity.^11^

Daily nutrient intake may crucially influence GV. In our study, no significant differences were found in the daily nutrient intake between the two groups, yet increased daily protein intake positively correlated with both AUCn and TIR, indicating improved intraday GV control. In contrast, higher fat intake per body weight was positively correlated with SD and negatively with AUCn and TIR, implying diminished glycemic stability. This aligns with Tettamanzi et al.'s findings that high‐protein diets can better manage insulin resistance and GV in morbidly obese, insulin‐resistant women compared to isocaloric Mediterranean diets,^31^ a notion also supported in type 1 diabetic children.^32^ The impact of fat intake on GV, however, remains debated. While some studies suggest a negative correlation between fat intake and GV,^33^ Blaychfeld‐Magnazi et al. observed that a low‐carbohydrate, high‐fat diet effectively reduces glycemic fluctuations in type 2 diabetes patients.^34^

Limited research has been undertaken to explore how the timing of basal insulin injections impacts GV and hypoglycemia frequency. Due to the relatively stable pharmacokinetic profile of Gla‐300 and Deg‐100, evenly distributed across 24 hours, there are no strict guidelines regarding their injection times in current practices. Prior studies have indicated that for type 1 diabetes patients, administering insulin glargine U100 either at dinner or bedtime, alongside fast‐acting analogues or regular human insulin, effectively and safely reduces HbA1c levels without significant differences in severe hypoglycemia between the two timings.^35^ Our study suggests that Deg‐100 administered HS may result in a higher LBGI than QD, indicating an increased risk of hypoglycemic events. In contrast, Gla‐300 administered QD showed significantly lower levels of short‐interval GV (CONGA1, CONGA2, and CONGA4) compared to HS administration. To deepen understanding, larger randomized controlled trials are warranted.

### 4.1 Strength and limitations

This study has several limitations. First, the patients needed to be under the two specific basal insulin analogues and be willing to undergo CGM, and keep a detailed food diary, which led to a relatively small sample size. While the relatively small sample size may limit the statistical power to detect more subtle differences, particularly in outcomes such as GV or hypoglycemia risk, this study serves as a pilot investigation to explore the impact of different insulin administration timings. Despite the sample size, we observed meaningful trends that provide preliminary insights and lay the groundwork for future, larger‐scale studies. Second, CGM data were collected through different devices, pooling metrics from different CGM devices. Third, the two brands of CGM manufacturers recommend the duration of CGM was 7 days, therefore, the number of days for CGM was shorter than the International Consensus. But we still can see the trend of TIR and FV according to the CGM record. Fourth, this was a cross‐sectional study and not a randomized trial. Fifth, the major nutrient components were self‐reported by participants; although rechecked and confirmed by a certified dietitian, there might still be deviation. Sixth, the daily activities of the participants were not regulated in this study, and we could not rule out the possibility of excessive exercises affecting GV.

The strengths of this study are as follows: the different effects of the two second‐generation basal insulin analogues on GV and their correlation with major nutrient components in real‐world clinical practice were studied, which was previously unclear. This is also the first study to examine this issue using CGM data from Taiwan.

## 5 CONCLUSIONS

In a real‐world study with type 1 diabetes patients, Gla‐300 showed the potential for better GV stability compared to Deg‐100. Additionally, when Deg‐100 was injected once daily in the morning (QD), it lowered the risk of hypoglycemia in the Deg‐100 group. In the case of Gla‐300, QD injections were also found to decrease GV compared to once‐daily bed‐time(HS) injections.

## CONFLICT OF INTEREST STATEMENT

The authors declare that they have no conflicts of interest.

## Supporting information


**Supplementary Table 1.** Demographic results of insulin degludec U100 and insulin glargine U300 users who received insulin injection at different time points.

**Supplementary Table 2.** Changes in HbA1c and insulin dose before and after CGM.
