# Correction to “Effects of Insulin Glargine U300 Versus Insulin Degludec U100 on Glycemic Variability, Hypoglycemia, and Diet Evaluated by Continuous Glucose Monitoring in Type 1 Diabetes: A Retrospective Cross‐Sectional Study”

**DOI:** 10.1002/kjm2.70090

**Published:** 2025-08-13

P.‐L. Tsai, C.‐H. Lin, Y.‐Y. Huang, H.‐Y. Chen, and Y.‐H. Lin, “Effects of Insulin Glargine U300 Versus Insulin Degludec U100 on Glycemic Variability, Hypoglycemia, and Diet Evaluated by Continuous Glucose Monitoring in Type 1 Diabetes: A Retrospective Cross‐Sectional Study,” *Kaohsiung Journal of Medical Sciences* 40, no. 12 (2024): 1086–1094, 10.1002/kjm2.12909

In the originally published version of this article, several values in *Table 2* under the parameter AUC180 (mg/dL) have now been corrected based on actual calculated data. The revised values are presented below:

All day period (00:00–24:00)

- Total study population: Original: 9223.37 [9223.37, 9223.37] → Corrected: 26912.16 [10416.24, 43408.08]
- Received Insulin Degludec U100:Original: 9223.37 [9223.37, 9223.37] → Corrected: 30754.45 [14939.64, 46569.25]
- Received Insulin Glargine U300:Original: 9223.37 [9223.37, 9223.37] → Corrected: 23069.87 [6415.61, 39724.12]
- *p* value: Original: 0.056 → Corrected: 0.662

Nocturnal period (00:00–06:00)

- Total study population: Original: 5558.3 [1933.44, 9223.37] → Corrected: 7244.28 [1106.85, 13381.70]
- Received Insulin Degludec U100: Original: 9223.37 [3925.69, 9223.37] → Corrected: 9024.04 [3476.24, 14571.84]
- Received Insulin Glargine U300: Original: 3943.23 [622.72, 7624.25] → Corrected: 5464.51 [–849.91, 11778.93]
- *p* value: Original: 0.033 → Corrected: 0.856

Diurnal period (06:00–24:00)

- Total study population: Original: 9223.37 [9223.37, 9223.37] → Corrected: 20090.49 [7535.13, 32645.84]
- Received Insulin Degludec U100:Original: 9223.37 [9223.37, 9223.37] → Corrected: 22007.39 [9739.55, 34275.23]
- Received Insulin Glargine U300:Original: 9223.37 [9211.25, 9223.37] → Corrected: 18173.58 [5315.32, 31031.84]
- *p* value: Original: 0.183 → Corrected: 0.440

In the originally published version of this article, the values for **AUC180 (mg/dL)** in *Table 4* under the parameter AUC180 (mg/dL) have now been corrected based on actual calculated data.

All day period (00:00–24:00)

- Degludec Bedtime Original: 9223.37 [9223.37, 9223.37] → Corrected: 20143.88 [13918.77, 27941.73]
- Degludec Morning Original: 9223.37 [9223.37, 9223.37] → Corrected: 20143.88 [13918.77, 27941.73]
- *p* value: Original: 0.081 → Corrected: 0.171
- Glargine Bedtime Original: 9223.37 [9223.37, 9223.37] → Corrected: 20143.88 [13918.77, 27941.73]
- Glargine Morning Original: 7844.25 [7489, —] → Corrected: 7844.25 [7666.63, 13894.68]

We apologize for this error. The authors confirmed that all results and conclusions of this article remain unchanged.

