# Supplementary material for: Effects of insulin glargine U300 versus insulin degludec U100 on glycemic variability, hypoglycemia, and diet evaluated by continuous glucose monitoring in type 1 diabetes: a retrospective cross‐sectional study
Source: Kaohsiung J Med Sci. 2024 Nov 26;40(12):1086–94. doi: 10.1002/kjm2.12909 (PMC11618557; doi:10.1002/kjm2.12909)
Supplement: Supplementary file 1 — Supplementary Table 1. Demographic results of insulin degludec U100 and insulin glargine U300 users who received insulin injection at different time points. [file KJM2-40-1086-s001.docx]

Supplementary Table 1. Demographic results of insulin degludec U100 and insulin glargine U300 users who received insulin injection at different time points

|  | Received Insulin Degludec U100 (n=20) | | | Received Insulin Glargin U300 (n=20) | | |
| --- | --- | --- | --- | --- | --- | --- |
|  | HS (n=13) | QD (n=7) | p | HS (n=16) | QD (n=3) | p |
| Age, years | 33.29 [26.88, 51.09] | 32.68 [32.47, 53.39] | 0.938 | 33.13 [28.86, 45.31] | 34.07 [25.74, -] | 1.000 |
| Sex, male, n (%) | 5 (38.4) | 1 (14.3) | 0.260 | 3 (18.8) | 1 (33.3) | 0.570 |
| Duration of CGM, days | 7 [6, 8] | 8 [7, 8] | 0.211 | 7 [7, 8] | 6 [6, -] | 0.109 |
| BW, kg | 64 [57.5, 68.85] | 53 [49, 58.4] | 0.030* | 57.95 [51.25, 70.25] | 59 [50.2, -] | 0.875 |
| BMI, kg/m2 | 23.44 [20.94, 27.38] | 22.33 [21.78, 22.81] | 0.211 | 22.49 [20.34, 25.84] | 20.42 [20.19, -] | 0.421 |
| Duration of disease, years | 11 [5, 21] | 14 [5, 29] | 0.817 | 13 [8, 20] | 19 [6, -] | 0.654 |
| Total daily insulin dose, U (Divided by body weight, U/kg) | \| 38 [31, 57.5] \| \| --- \| \| (0.6 [0.53, 0.93]) \| | \| 39 [31, 80] \| \| --- \| \| (0.76 [0.62, 1.28]) \| | 0.757  (0.183) | \| 45.5 [33.75, 58] \| \| --- \| \| (0.76 [0.54, 1.07]) \| | 55 [37, -]  (0.88 [0.74, -]) | 0.712  (0.559) |
| Basal daily insulin dose, U (Divided by body weight, U/kg) | \| 15 [11.5, 20.5] \| \| --- \| \| (0.23 [0.18, 0.33]) \| | \| 19 [13, 30] \| \| --- \| \| (0.34 [0.27, 0.51]) \| | 0.485  (0.046*) | \| 15 [10, 16.75] \| \| --- \| \| (0.25 [0.18, 0.3]) \| | \| 25 [7, -] \| \| --- \| \| (0.41 [0.14, -]) \| | 0.559  (0.487) |
| HbA1c before CGM, % (mmol/mol) | \| 8.5 [7.75, 9.7] \| \| --- \| \| (69.41 [61.21, 82.52]) \| | \| 8 [7.9, 9] \| \| --- \| \| (63.94 [62.85, 74.87]) \| | 0.393 | \| 7.9 [7.2, 9] \| \| --- \| \| (62.85 [55.2, 74.87]) \| | \| 7.7 [6.5, -] \| \| --- \| \| (60.66 [47.55, -]) \| | 0.574 |
| Nutrient composition per day, % (g) | | | | | | |
| Carbohydrate, % (g) | \| 47.55 [45, 52.7] \| \| --- \| \| (169.68 [139.18, 207.12]) \| | \| 45.86 [39.78, 50.22] \| \| --- \| \| (154.6 [144.38, 194.8]) \| | 0.485  (0.817) | \| 44.94 [41.07, 51.41] \| \| --- \| \| (164.48 [119.76, 182.5]) \| | \| 45.03 [36.08, -] \| \| --- \| \| (156.97 [103, -]) \| | 0.487  (1.000) |
| Protein, % (g) | \| 15.27 [13.88, 17.52] \| \| --- \| \| (59.12 [48.33, 66.85]) \| | \| 15.38 [14.38, 17.9] \| \| --- \| \| (66 [48.6, 67]) \| | 0.938  (0.938) | \| 16.48 [14.77, 18.34] \| \| --- \| \| (53.19 [43.68, 71.19]) \| | \| 16.5 [16.03, -] \| \| --- \| \| (57 [54.53, -]) \| | 0.634  (0.559) |
| Fat, % (g) | \| 37.23 [31.97, 39.86] \| \| --- \| \| (63.78 [44.32, 72]) \| | \| 37.56 [34.4, 41.8] \| \| --- \| \| (62.57 [47, 78.8]) \| | 0.643  (0.817) | \| 35.94 [32.92, 42.05] \| \| --- \| \| (50.68 [41.38, 71.74]) \| | \| 38.47 [37.25, -] \| \| --- \| \| (55 [54.67, -]) \| | 0.359  (0.359) |
| Average daily carbohydrates per body weight, g/kg | 2.63 [2.49, 2.8] | 2.92 [2.86, 3.05] | 0.067 | 2.65 [2.2, 3.05] | 2.66 [1.61, -] | 1.000 |
| Average daily protein per body weight, g/kg | 0.92 [0.75, 1.06] | 1.11 [0.92, 1.19] | 0.157 | 0.98 [0.73, 1.09] | 0.92 [0.89, -] | 1.000 |
| Average daily fat per body weight, g/kg | 1 [0.74, 1.03] | 1.07 [0.92, 1.51] | 0.211 | 0.95 [0.71, 1.2] | 0.93 [0.86, -] | 0.712 |
| Average daily calories, kcal | 1406.66 [1209.52, 1716.65] | 1458.73 [1215, 1794.8] | 0.877 | 1280.04 [1142.81, 1709.28] | 1338 [1135, -] | 0.793 |
| Average daily calories per body weight, kcal/kg | 22.74 [19.47, 25.1] | 25.92 [23.59, 29.45] | 0.046* | 23 [18.01, 28.34] | 22.68 [17.73, -] | 0.875 |

Data of age, duration of CGM, body weight, body mass index, disease duration, insulin dose, and nutrient composition are presented as median [Q1, Q3].

Continuous variants were analyzed by Mann–Whitney U test, and nominal variants were analyzed by McNemar’s chi-square test.

Abbreviations: CGM, continuous glucose monitoring; BMI, body mass index; HbA1c, glycated hemoglobin; Q, quartile; BW, body weight; HS, once daily in the evening; QD, once daily in the morning
