# Supplementary material for: Effects of insulin glargine U300 versus insulin degludec U100 on glycemic variability, hypoglycemia, and diet evaluated by continuous glucose monitoring in type 1 diabetes: a retrospective cross‐sectional study
Source: Kaohsiung J Med Sci. 2024 Nov 26;40(12):1086–94. doi: 10.1002/kjm2.12909 (PMC11618557; doi:10.1002/kjm2.12909)
Supplement: Supplementary file 2 — Supplementary Table 2. Changes in HbA1c and insulin dose before and after CGM. [file KJM2-40-1086-s002.docx]

Supplementary Table 2. Changes in HbA1c and insulin dose before and after CGM

|  | Received Insulin Degludec U100  (n=20) | | | Received Insulin Glargin U300  (n=20) | | |
| --- | --- | --- | --- | --- | --- | --- |
|  | before CGM | post 3 month | p | before CGM | post 3 month | p |
| HbA1c, % (mmol/mol) | \| 8.35 [7.9, 9.3] \| \| --- \| \| (67.77 [62.85, 78.15]) \| | 8.1 [7.83, 8.95]  (65.03 [62.03, 74.32]) | 0.386  (0.334) | \| 7.9 [7.2, 9] \| \| --- \| \| (62.85 [55.2, 74.87]) \| | \| 7.7 [6.7, 8.5] \| \| --- \| \| (58.48 [49.73, 69.13]) \| | 0.111  (0.102) |
| Total daily insulin dose, U (Divided by body weight, U/kg) | \| 38.5 [31.25, 60] \| \| --- \| \| (0.64 [0.55, 1.12]) \| | \| 39.5 [30.75, 54.75] \| \| --- \| \| (0.7 [0.53, 1.09]) \| | 0.308  (0.333) | \| 46.5 [36, 57.5] \| \| --- \| \| (0.76 [0.62, 0.93]) \| | \| 47.5 [30.75,56.25] \| \| --- \| \| (0.79 [0.57, 0.95]) \| | 0.944  (0.917) |
| Basal daily insulin dose, U (Divided by body weight, U/kg) | \| 18.5 [12, 20.75] \| \| --- \| \| (0.29 [0.22, 0.34]) \| | 19 [12, 20]  (0.28 [0.21, 0.35]) | 0.233  (0.310) | \| 15 [10, 21] \| \| --- \| \| (0.26 [0.18, 0.32]) \| | \| 16 [11.25, 20.75] \| \| --- \| \| (0.28 [0.2, 0.34]) \| | 0.858  (0.859) |

Data of HbA1c and insulin dose are presented as median [Q1, Q3].

Continuous variants were analyzed by Mann–Whitney U test

Abbreviations: CGM, continuous glucose monitoring; HbA1c, glycated hemoglobin; Q, quartile
